# miR-19b-3p/PKNOX1 Regulates Viral Myocarditis by Regulating Macrophage Polarization

**DOI:** 10.3389/fgene.2022.902453

**Published:** 2022-06-24

**Authors:** Chen Jiahui, Zheng Jiadai, Zheng Nan, Zhou Rui, Huang Lipin, He Jian, Zhu Wenzong, Zhang Riyuan

**Affiliations:** ^1^ Yining Hospital Affiliated to Wenzhou Medical University, Wenzhou, China; ^2^ Zhejiang Chinese Medical University, Hangzhou, China; ^3^ Wenzhou Hospital of Traditional Chinese Medicine Affiliated to Zhejiang Chinese Medical University, Wenzhou, China

**Keywords:** left ventricular short axis shortening rate, left ventricular ejection fraction, viral myocarditis, Coxsackie virus B3, macrophages

## Abstract

**Objective:** The purpose of this study was to study the role and mechanism of miR-19b-3p in regulating myocardial inflammation and injury of viral myocarditis in viral myocarditis induced by Coxsackievirus B3 (CVB3). A CVB3 infection mouse model was established, the survival rate of mice was recorded after different treatments, cardiac function was detected, the degree of myocardial inflammatory infiltration and injury was detected by immunohistochemical and biochemical analyses, miR-19b-3p and PKNOX1 expression in cardiac tissue and cardiac infiltrating macrophages was detected using RT-PCR, and isolated mouse bone marrow-derived macrophages and the differentiation of macrophages after different transfections were detected. Finally, the binding of miR-19b-3p and PKNOX1 was verified by the dual luciferase reporter gene. The results showed that the expression of miR-19b-3p was significantly downregulated in the cardiac tissue and infiltrating macrophages of CVB3-infected mice, while the expression of PKNOX1 was upregulated. Upregulation of miR-19b-3p has protective effects against CVB3-induced myocardial injury in mice, such as weight gain, prolonged survival, increased left ventricular ejection fraction and left ventricular short axis shortening, reduced inflammation, creatine kinase isoenzyme (CK)-MB, and lactate dehydrogenase (LDH), and aspartate aminotransferase (AST) levels decreased, while interferon-γ and interleukin-6 (IL-6) increased, and the M2/M1 cell ratio was upregulated. In conclusion, miR-19b-3p can regulate macrophage polarization by targeting PKNOX1, and has a protective effect against CVB3-induced inflammation and myocardial injury.

## Introduction

Viral myocarditis (VM) is an inflammatory disease, and Coxsackie virus B3 (CVB3) is considered to be the most common pathogen of VM (Zhang et al., 2016; Wang et al., 2020a; Xia et al., 2020). Inflammation is the main pathological factor in the pathogenesis of VM, but its specific regulatory mechanism is still unclear (Wang et al., 2020b). Macrophages, the main inflammatory cell subset, can be enriched in cardiac tissue 3 days after CVB3 infection. Depending on the cardiac microenvironment, macrophages can polarize into a classically activated M1 phenotype or an alternately activated M2 phenotype. M1 macrophages are induced by lipopolysaccharide and interferon-γ (IFN-γ), and promote the development of myocardial inflammation by producing inflammatory cytokines (Zhao et al., 2019). In contrast, M2-type macrophages induced by interleukin (IL)-4 or IL-13 can secrete anti-inflammatory cytokines associated with tissue repair. Related studies have shown that intravenous injection of M1 macrophages *in vitro* can promote the development of myocarditis in mice, while M2 macrophages can significantly reduce myocardial inflammation in mice by regulating the distribution of local cytokines (Li et al., 2020a). However, the specific regulatory mechanism of macrophage polarization during VM inflammation is not fully understood. Recent studies have pointed out that the expression of PKNOX1 in the serum of patients with systemic inflammatory diseases is upregulated, and miR-935 inhibitors can target the destruction of HOX/PBX/MEIS/PKNOX interaction to inhibit the release of inflammatory factors and alleviate lipopolysaccharide-induced acute lung injury in mice (Fan et al., 2019). Related studies have shown that miR-19b-3p can protect diet-induced adipose tissue inflammation by regulating macrophage polarization, inhibiting canonical inflammatory pathways, and enhancing other anti-inflammatory responses (Yang et al., 2019). Preliminary experiments in this study also showed that miR-19b-3p could target the expression of PKNOX1 in cardiomyocytes. Therefore, this manuscript combines the potential roles of miR-19b-3p and PKNOX1 in inflammatory responses and macrophage polarization, speculating that miR-19b-3p may regulate CVB3 infection-induced myocardial inflammation through PKNOX1, and trying to explore its regulatory effect on macrophage polarization.

## Materials and Methods

### Laboratory Animal

Forty male BALB/c mice aged 6–7 weeks were purchased from Shanghai Jisijie Laboratory Animal Company, weighing (22.7 ± 2.94) g, and raised in a specific pathogen-free environment with a 12-h light/dark cycle. All experimental steps comply with animal ethics requirements (ethical approval number Z20191106).

### Main Reagents and Instruments

The pLL3.7 vector was purchased from Chongqing Youbao Biotechnology Co., Ltd. (VT2204), TBST buffer (T196393) and phosphate buffer (P301981) were all purchased from Shanghai Aladdin Reagent Co., Ltd., Rabbit anti-mouse PKNOX1 monoclonal antibody (ab182858), horseradish peroxidase-labeled immunoglobulin conjugate-rabbit anti-mouse IgG conjugate (ab233006) were all purchased from Abcam, United Kingdom. SuperScript IV First Strand cDNA Synthesis Kit (K1612), SuperScript IV Reverse Transcriptase (18064071) and Trizol (A33253) were all purchased from Thermo Fisher Scientific. RPMI 1640 medium (R8758) was purchased from Sigma-Aldrich, United States. The low-speed centrifuge (TDZ4B-WS) was purchased from Shanghai Bain Biotechnology Co., Ltd. CO_2_ incubator (BC-J160S) and ultra-clean workbench (SW-CJ-2FD) were purchased from Shanghai Boxun Co., Ltd. 4°C centrifuge (centrifuge 5415R) and RT-PCR instrument (realplex) were purchased from Eppendorf, Germany. The inverted fluorescence electron microscope (DMI3000B) was purchased from Leica, Germany, and the electrophoresis apparatus (EPS300) was purchased from Shanghai Tianneng Technology Co., Ltd. A microplate reader (GloMax^®^ Discover) was purchased from Promega Corporation, United States. An inverted microscope (GX41) was purchased from Olympus Company in Japan, and a flow cytometer (CytoFLEX) was purchased from Beckman Coulter Company in the United States.

### Animal Grouping and Handling

Forty mice were randomly divided into four groups, with 10 mice in each group, namely, the CVB3 group, miR-19b-3p group, CVB3+NC group, and control group. The CVB3 group, miR-19b-3p group, and CVB3+NC group of mice were intraperitoneally injected with 6 × 10^3^ CVB3-infected HeLa cells to establish a VM mouse model (Fan et al., 2019), and the control group received intraperitoneal injection of PBS only. On the first and third days after modeling, mice in the miR-19b-3p group were intraperitoneally injected with the lentiviral vector pLL3.7-miR-19b-3p (50 μg/kg), and mice in the CVB3+NC group were intraperitoneally injected with an equal volume of empty vector pLL3. Four groups of mouse heart tissues were collected on the seventh day after modeling for follow-up experiments.

Echocardiography left ventricular ejection fraction (LVEF) and left ventricular short axis shortening rate (LVFS) were measured by the echocardiography system (Li et al., 2020a) according to the operating manual.

### Histopathology and Myocarditis Severity Assessment

On the seventh day after infection with CVB3, cardiac tissue was collected, fixed, embedded, and sectioned for hematoxylin–eosin (H&E) staining. The severity of myocarditis was assessed by the percentage of the area with inflammation in the heart section to the size of the entire section.

### Enzyme-Linked Immunosorbent Assay

After the mice were killed by cervical dislocation, the hearts were removed, the tissue pieces were chopped as soon as possible with small ophthalmic scissors, and the hearts were made into 10% myocardial tissue normal saline homogenate with a tissue masher at 10,000–15,000 r/min and centrifuged for 14 min; 0.5 ml of the supernatant was taken for later use; the detection of IFN-γ, IL-6, IL-10, CK-MB, LDH, and AST in the supernatant of cardiac homogenate was carried out using the corresponding cytokine ELISA kit (R&D system) according to the instructions of the reagent manufacturer.

### Cell Isolation and Culture

The heart tissue was taken and cut into small pieces of 1 mm^3^, digested with 0.1% type II collagenase and 0.01% hyaluronidase for 2 h, and the inflammatory cells were isolated from single cells by density gradient separation; macrophages isolated by FACS stained with FITC-labeled anti-F4/80 monoclonal antibody. The femur and tibia were dissected from normal mice, the bone marrow was flushed, and bone marrow-derived macrophages (BMDM) were isolated. The cells were cultured with a complete medium for 7 days and seeded in 6-well plates (1×10^6^/well). Polarization induction was performed with RPMI 1640 + 5% fetal bovine serum, 10 ng/ml lipopolysaccharide +20 ng/ml IFN-γ (M1 polarized), or 20 ng/ml IL-4 (M2 polarized).

### Flow Cytometry Analysis

DMEM single cell suspensions were stained with FITC-labeled antibodies against CD86 and CD206 following the reagent manufacturer’s instructions, cell fluorescence was measured by FACS, and data were analyzed using FlowJo software.

### Cell Transfection

MiR-19b-3p mimics, mimic controls, plasmids, or blank plasmids were transfected and grown with Lipofectamine 2000 to 70% confluent BMDM, the medium was changed 6 h after transfection, and 24 h after transfection, cells were exposed to macrophage-polarized culture conditions for 24 h.

### Qualitative Real-Time Polymerase Chain Reaction

Extraction of total RNA from cardiac tissue and cells was performed using Trizol reagent. Reverse transcription using the miRNA First-Strand cDNA Synthesis Kit, qRT-PCR, was performed using TaqMan microRNA analysis and normalized to the expression of U6, and the relative expression was calculated by the 2^−ΔΔCt^ method (Table 1).

**TABLE 1:** Sequences of primers used in PCR.

Gene	Forward PCR primer	Reverse PCR primer
U6	AGA​CAT​GGA​CGT​GGT​GAA​TCA	ACT​CTC​CGT​CTT​GTT​GGC​AC
MiR-19b-3p	TTC​TCC​TGT​TTT​ATG​GGG​ACT​GA	CCC​TAC​CCG​AAA​TGC​ACT​GTA
PKNOX1	CCA​TCA​GCA​TAG​GTG​GAC​TTT​T	TGG​TGT​TGT​ATA​ACT​GCA​CAG​C

### Luciferase Reporter Assay

BMDM cells were seeded in 96-well plates (2 × 10^3^/well); after 24 h, pGL3-PKONX1 3,-UTR (WT) or pGL3-PKONX1 3′-UTR Mut plasmid (contains wild-type or mutant miR-19b-3p binding sites), miR-19b-3p mimetic or miR-19b-3p-NC co-transfected cells with PRL-SV40 renal luciferase vector with liposome 2000, after 8 h, the firefly and nephron luciferase activities were measured by the dual luciferase assay, and the relative luciferase activity was expressed by the nephron/firefly luciferase activity.

### Western Blot

Myocardial tissue and total cell protein were lysed with RIPA extract, and the intracellular protein concentration was determined by the BCA method, protein cleavage products were separated by SDS-PAGE and electro-transferred to a nitrocellulose membrane; after blocking with 5% skim milk for 1 h, they were incubated with anti-PKNOX1 antibody (1:1000) and anti-GAPDH antibody (1:1000) overnight at 4°C, respectively; then they were incubated with the corresponding secondary antibody (1:10000) at 37°C for 2 h, and finally, the immunoblot was observed with an infrared imaging system.

### Statistical Analysis

Data analysis was performed using GraphPad Prism 5 statistical software, and all data are expressed as mean ± SEM. One-way ANOVA was used for comparison among multiple groups, and the LSD-t test was used for pairwise comparison between groups. *p* value <0.05 was considered statistically significant.

## Results

### Expression of miR-19b-3p and PKNOX1 in Mice in Different Treatment Groups

**FIGURE 1:**
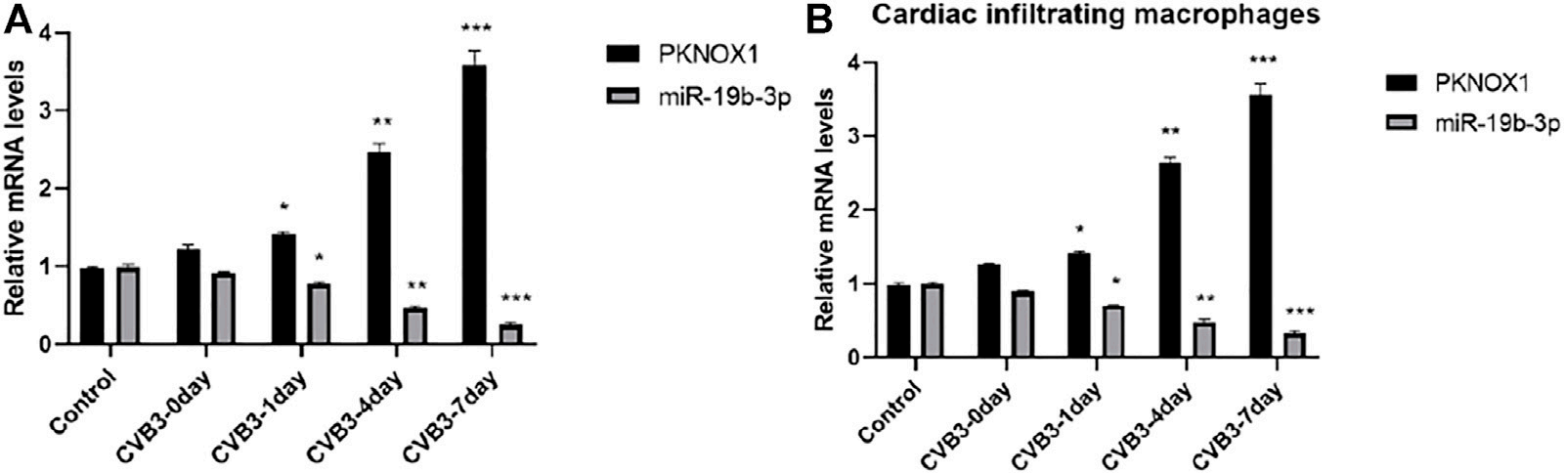
Comparison of miR-19b-3p and PKNOX1 expression after different treatments. **(A)** PCR detection of miR-19b-3p and PKNOX1 expression in mouse heart tissue and **(B)** PCR detection of miR-19b-3p and PKNOX1 expression in cardiac infiltrating macrophages. Compared with the control group, ∗*p* < 0.05, ∗∗*p* < 0.01, ∗∗∗*p* < 0.001.

PCR detection showed that compared with the control group or before CVB3 infection, the expression of miR-19b-3p in myocardial tissue of CVB3 group mice was significantly decreased on the seventh day after CVB3 infection, and its expression decreased in a time-dependent manner; the expression of PKNOX1 increased in a time-dependent manner, suggesting that miR-19b-3p and PKNOX1 may be involved in the pathogenesis of CVB3 myocarditis (Figure 1A); in addition, compared with the PBS group, the expression of miR-19b-3p was significantly decreased, and the expression of PKNOX1 was increased in the cardiac infiltrated macrophages of the CVB3 group of mice; it is suggested that miR-19b-3p and PKNOX1 may play a regulatory role in CVB3-induced VM by regulating macrophage activity (Figure 1B).

### Expression of Inflammatory Cytokines in Mice in Different Treatment Groups

**FIGURE 2:**
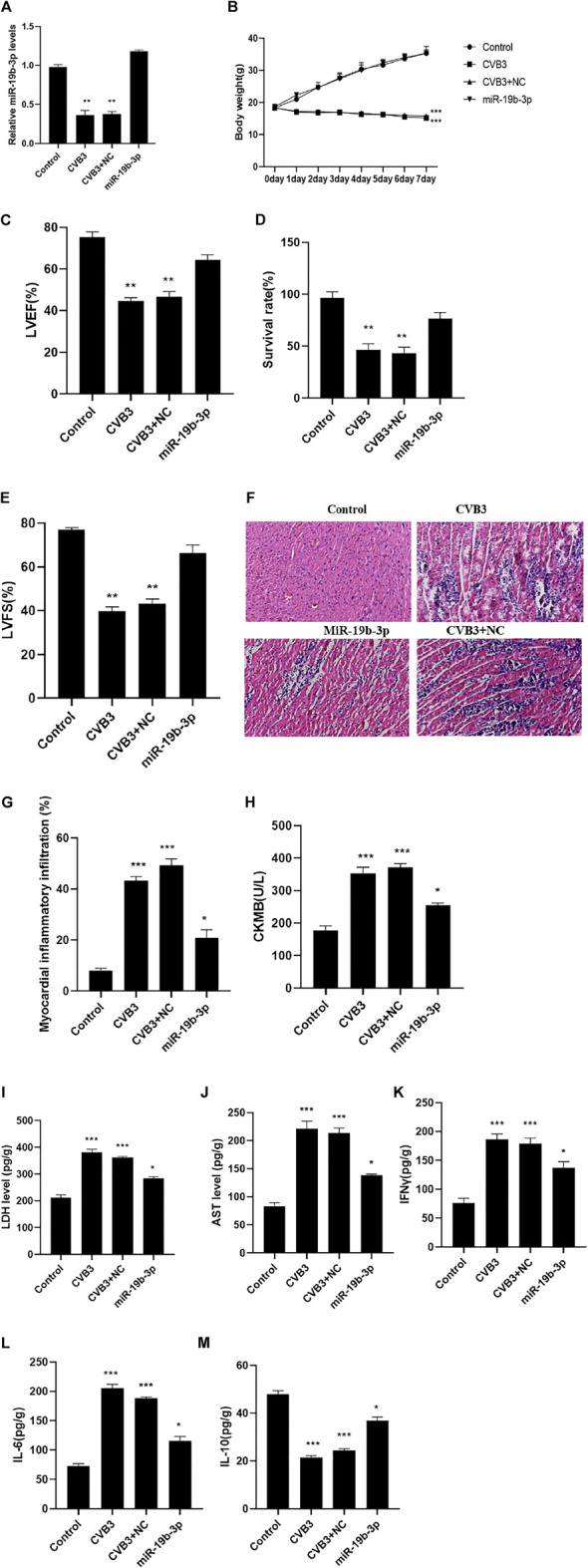
(Continued).

PCR detection showed that compared with the control group, the expression of miR-19b-3p in the CVB3 group was significantly reduced, the body weight of the mice continued to decrease, and the survival rate, LVEF, and LVFS were significantly reduced. Compared with the CVB3 group, the expression of miR-19b-3p in the myocardial tissue of mice was significantly increased, and the survival rate, LVEF, and LVFS were significantly increased (Figures 2A–E). Further immunohistochemical and biochemical analyses showed that compared with the CVB3 group, the myocardial inflammation in the miR-19b-3p group was significantly reduced (Figure 2F); the levels of CK-MB, LDH, and AST were significantly reduced; inflammatory cytokines (IFN-γ and IL-6) were significantly reduced; and the release of anti-inflammatory cytokine (IL-10) was significantly upregulated (Figures 2G–M).

### M1/M2 Polarization of Macrophages Under Different Treatments

**FIGURE 3:**
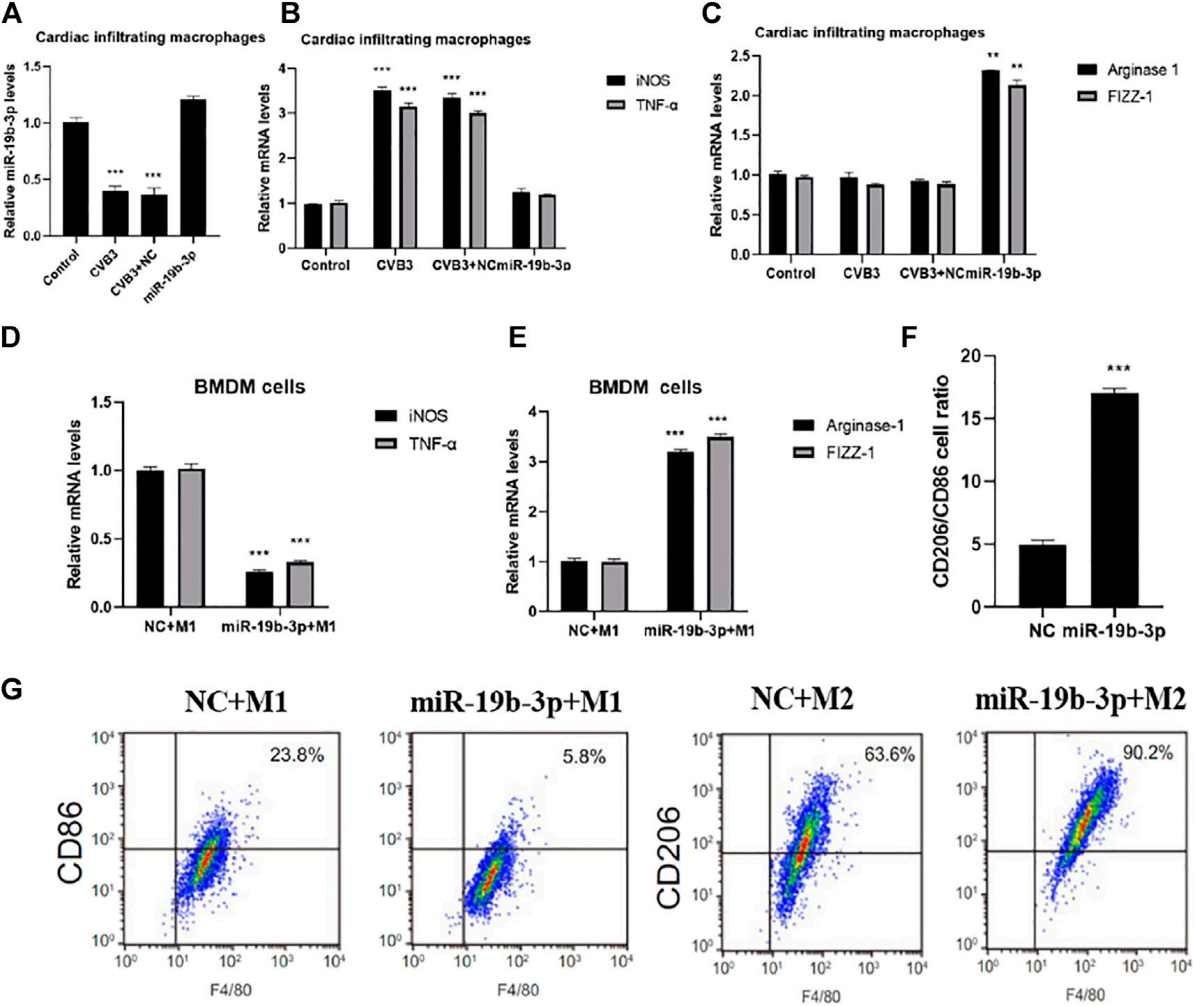
Comparison of M1/M2 polarization of macrophages under different treatments. PCR detection of miR-19b-3p **(A)**, inducible nitric oxide synthase and tumor necrosis factor-α **(B)**, arginase-1 and Fizz-1 **(C)** expressions in cardiac infiltrating macrophages, PCR detection of **(D)** inducible nitric oxide synthase and TNF-α5 **(D)**, arginase-1 and FIZZ-1 **(E)** in BMDM cells under different polarization conditions, **(F)** CD206/CD86 cell ratio detected by flow cytometry, and **(G)** representative images of flow cytometry assays.∗*p* < 0.05, ∗∗*p* < 0.01, ∗∗∗*p* < 0.001.

The expression of miR-19b-3p was significantly downregulated in CVB3-infected mouse cardiac infiltrating macrophages and was significantly upregulated in the miR-19b-3p group (Figure 3A). The Western blot analysis showed that the expression of macrophage M1 markers (iNOS and TNF-α) was significantly increased in the CVB3 group compared with the control group, and there was no significant difference in the expression of M2 markers (arginase-1 and FIZZ-1) (Figures 3B,C); compared with the CVB3 group, the expression of iNOS and TNF-α was significantly decreased in the miR-19b-3p group, the expressions of arginase-1 and FIZZ-1 were significantly increased, and it is suggested that miR-19b-3p can inhibit M1 polarization and enhance M2 polarization *in vivo*, thereby having a protective effect on CVB3-induced myocarditis.

*In vitro* transfection showed that miR-19b-3p was significantly overexpressed in BNDM cells after miR-19b-3p mimic transfection (Figure 3D), and flow cytometry showed that the expressions of iNOS and TNF-α were significantly downregulated in the miR-19b-3p + M1 group compared with the NC + M1 group, the expression of arginase-1 and FIZZ-1 was significantly increased (Figures 3E,F), and further flow cytometry detection showed that the overexpression of miR-19b-3p inhibited the production of the significant M1 macrophage marker CD86 and enhanced the expression of the M2 macrophage marker CD206 (Figures 3G,H), confirming that miR-19b-3p plays an important role in macrophage polarization by promoting M2 and suppressing M1 isoforms.

### Activity Status of Luciferase Under Different Treatments

**FIGURE 4:**
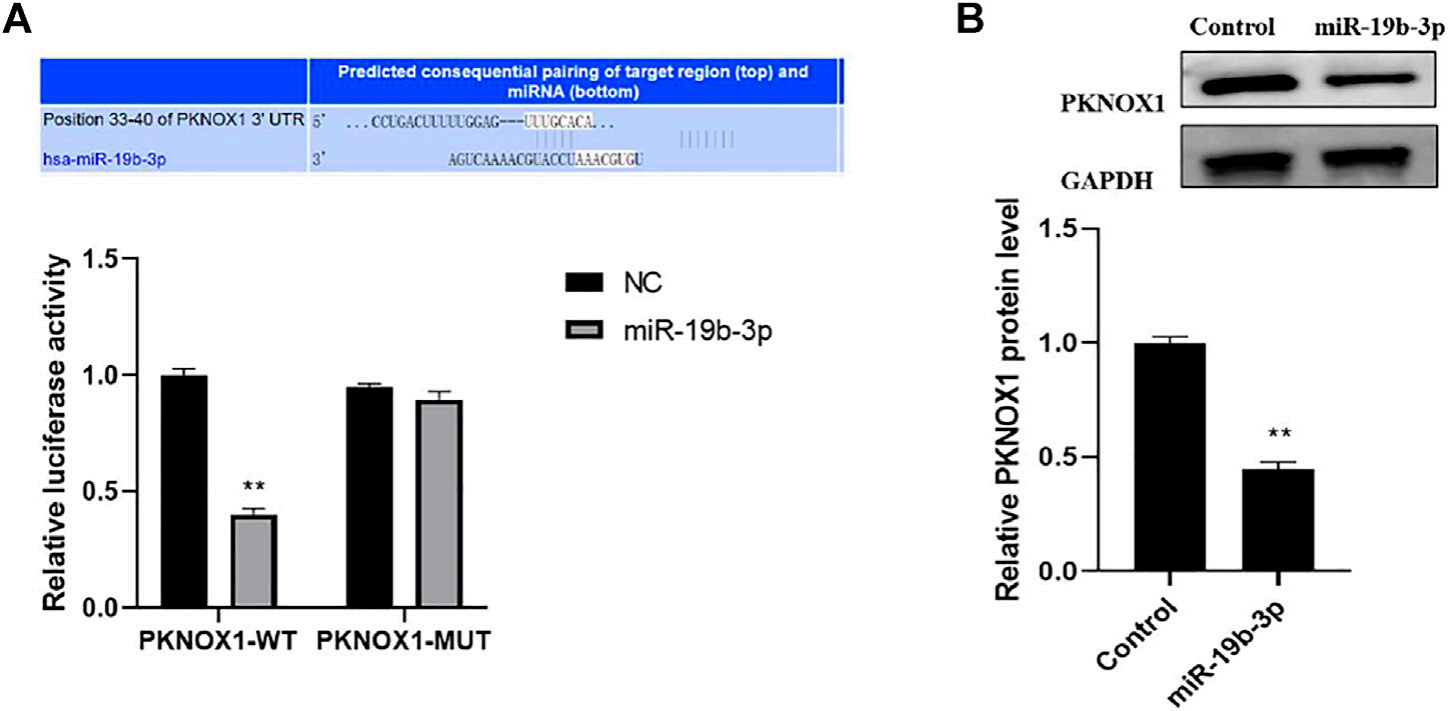
Comparison of luciferase activity under different treatments. **(A)** Bioinformatic analysis prediction and dual luciferase reporter gene validation of the binding of miR-19b-3p to PKNOX1 3′-UTR. **(B)** Western blot was used to detect the expression of PKNOX1 protein in BMDM after different transfections. Compared with the control group,∗*p* < 0.05, ∗∗*p* < 0.01, ∗∗∗*p* < 0.001.

MiR-19b-3p target genes were screened using target gene prediction algorithms (including Target Scan) and then validated with luciferase reporter experiments, The results showed that miR-19b-3p overexpression inhibited the luciferase activity of the PKNOX13′-UTR construct containing the miR-19b-3p binding site. In contrast, mutants containing miR-19b-3p binding sites did not show significant changes in luciferase activity (Figure 4A), and the Western blot analysis showed that overexpression of miR-19b-3p significantly inhibited the protein expression of PKNOX1 (Figure 4B); this indicated that miR-19b-3p could inhibit its expression by targeting the 3,UTR of PKNOX1.

### Status of Macrophage Polarization After Different Transfections

**FIGURE 5:**
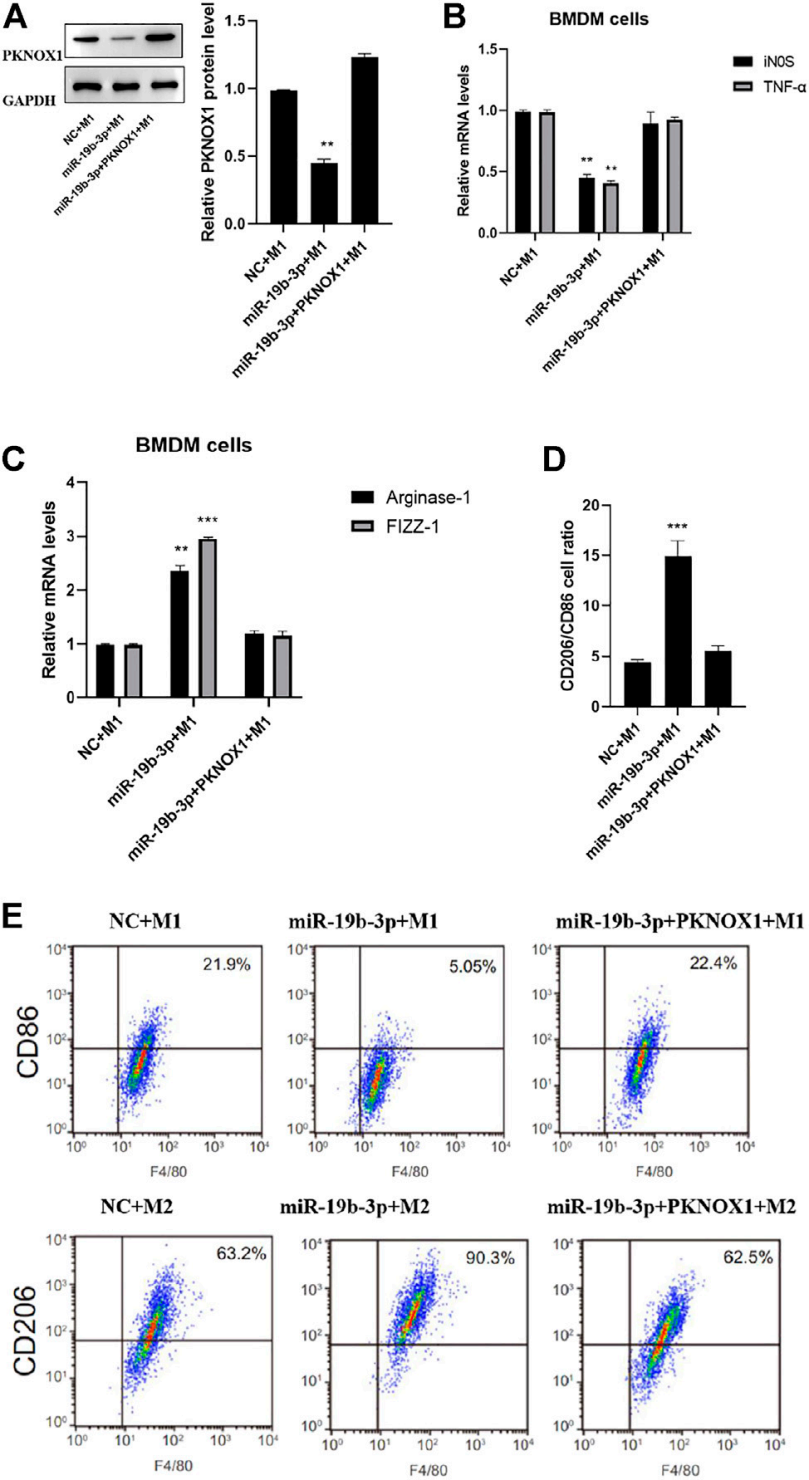
Comparison of the polarization status of macrophages after different transfections. Western blot analysis of **(A)** PKNOX1, qRT-PCR detection **(B)** expression of inducible nitric oxide synthase and tumor necrosis factor-α after different transfections. **(C)** The expression of arginase-1 and FIZZ-1 mRNA was detected by qRT-PCR. **(D)** Detection of CD206/CD86 cell ratio by flow cytometry and **(E)** representative images of flow cytometry assays. ∗*p* < 0.05, ∗∗*p* < 0.01, ∗∗∗*p* < 0.001.

PCR detection confirmed that compared with the miR-19b-3p group, the expression of PKNOX1 in the miR-19b-3p + PKNOX1 group was significantly upregulated (Figure 5A). PCR and flow cytometry assays showed that the mRNA levels of iNOS and TNF-α were significantly increased in the miR-19b-3p + PKONX11 + M1 group compared with the miR-19b-3p + M1 group (Figure 5B). Compared with the miR-19b-3p + M2 group, the up-regulated expressions of arginase-1 and FIZZ-1 induced by the overexpression of miR-19b-3p in the miR-19b-3p + PKONX11 + M2 group were significantly inhibited (Figures 5C–E). The flow cytometry analysis showed that PKNOX1 overexpression vector could reverse the miR-19b-3p-induced M2-type differentiation of macrophages. This indicated that PKNOX1 could partially mediate the effect of miR-19b-3p on macrophage activation.

## Discussion

The pathogenesis of CVB3-induced myocarditis remains unclear. miR-19b-3p was previously reported to be associated with various human cancers, metabolic disorders, cardiovascular diseases, and inflammatory diseases (Zhu et al., 2019). This study aimed to investigate the effect and mechanism of miR-19b-3p on CVB3-induced myocardial inflammation and injury. In this study, a mouse model of VM was established using CVB3-infected mice. In CVB3-infected mouse heart tissue, the expression of miR-19b-3p was decreased in a time-dependent manner, while the expression of PKNOX1 was significantly upregulated, and it was negatively correlated with the expression of miR-19b-3p, suggesting that the downregulation of miR-19b-3p and possibly the upregulation of PKNOX1 expression may be closely related to the occurrence of CVB3-induced VM.

Previous studies have shown that miR-19b-3p has anti-inflammatory and cardioprotective effects (Gou et al., 2018). In this study, pLL3.7-miR-19b-3p was injected intraperitoneally on days 1 and 3 after CVB3 infection to upregulate the expression of miR-19b-3p. The results showed that overexpression of miR-19b-3p *in vivo* can protect mice from CVB3-induced VM, and manifested as increased body weight, prolonged survival time, increased LVEF and LVEF, decreased inflammation, decreased levels of CKMB, LDH, and AST, and the production of IFN-γ, IL-6, and IL-10 reduce; it suggested that CVB3-induced myocarditis was significantly relieved, showing that miR-19b-3p could significantly alleviate CVB3-induced myocarditis. Research shows (Li et al., 2020b; Cao et al., 2020) virus-induced immune responses play an important role in myocardial injury during VM. Myocardial macrophages, as the earliest infiltrating inflammatory cells, participate in the immune response of VM, and different macrophage polarizations may play opposite roles in the inflammatory response. According to reports (Chen et al., 2017), M1 macrophages exacerbate myocardial inflammation, while M2 macrophages reduce myocardial inflammation. In the present study, CVB3 infection promoted the polarization of the M1 phenotype, along with a decrease in the transcriptional level of miR-19b-3p, after isolation of differently treated mouse heart-infiltrating macrophages, while overexpression of miR-19b-3p significantly promoted M2 and inhibited M1 subtype; *in vitro* analysis of BMDMs further confirmed the regulation of miR-19b-3p on macrophage polarization. Literature reports (Elton et al., 2013; Zhang et al., 2018; Pu et al., 2019) silencing miR-155 attenuates cardiac injury and dysfunction in VM by promoting macrophage M2 phenotype polarization. In the present study, the results confirmed that miR-19b-3p can also inhibit M1 polarization and enhance M2 polarization, thereby protecting against CVB3-induced myocarditis. miR-19b-3p regulates inflammation and infection by targeting many targets, including granzyme B, an activator of signaling and transcription 3, and PKNOX1. In this study, PKNOX1 was identified as a direct target of miR-19b-3p by bioinformatics prediction analysis and luciferase reporter gene analysis, and Western blot analysis further confirmed that miR-19b-3p can target the expression of PKNOX1. Further rescue experiments revealed that overexpression of PKNOX1 partially reversed the polarized phenotype regulated by overexpression of miR-19b-3p and manifested as marked changes in macrophage markers. These results suggest that PKNOX1 may partially mediate the effect of miR-19b-3p on macrophage activation.

In conclusion, the results of this study show that miR-19b-3p has a protective effect on CVB3-induced VM by targeting PKNOX1 to regulate macrophage polarization; however, the mechanism of action of miR-19b-3p in VM still needs further study.

## Data Availability

The original contributions presented in the study are included in the article/Supplementary materials, further inquiries can be directed to the corresponding author/s.
