# Supplementary figures and images for: miR-19b-3p/PKNOX1 Regulates Viral Myocarditis by Regulating Macrophage Polarization
Source: Front Genet. 2022 Jun 24;13:902453. doi: 10.3389/fgene.2022.902453 (PMC9264346; doi:10.3389/fgene.2022.902453)

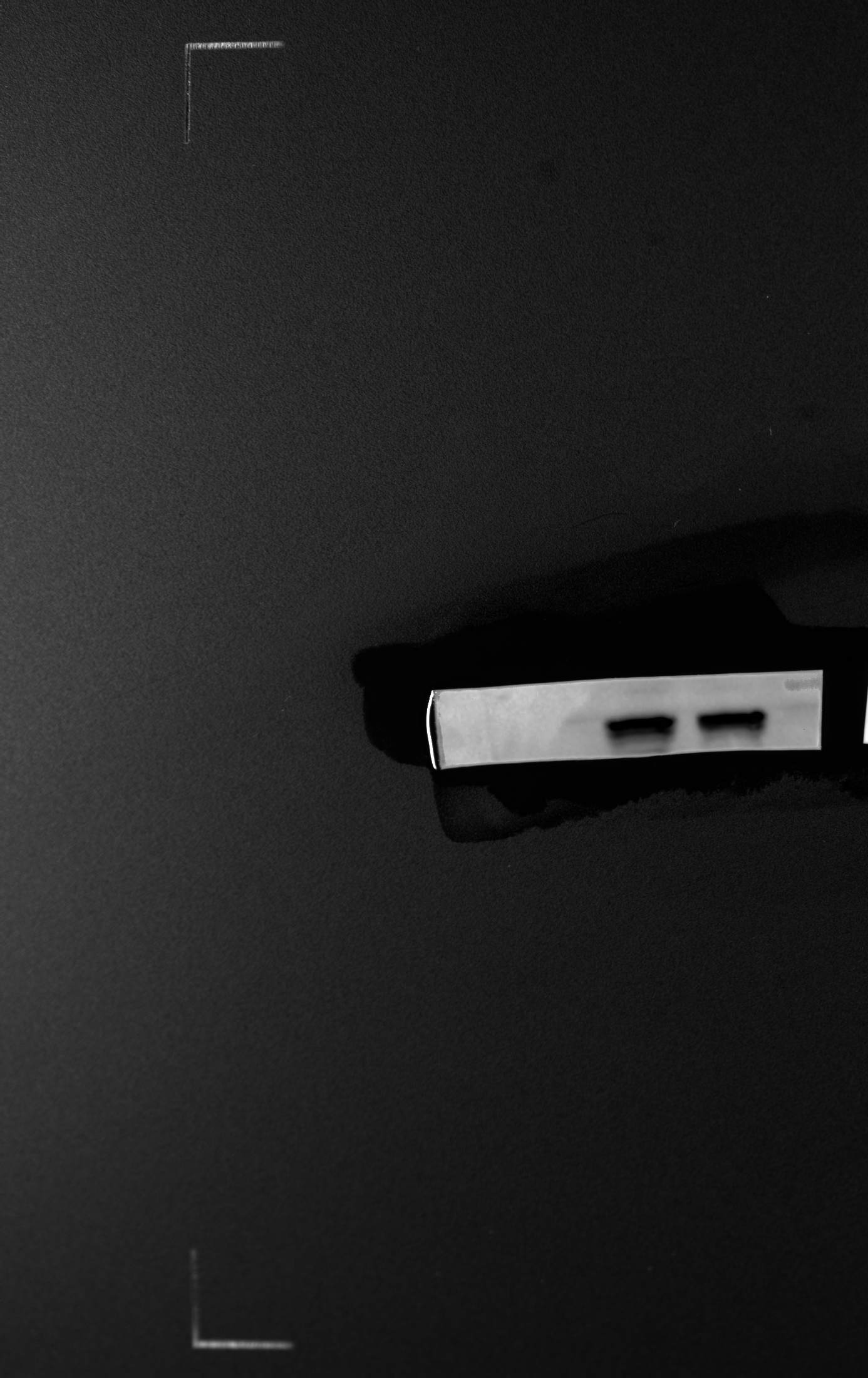

Supplement: Supplementary file 1 [file DataSheet1.zip › ╩2╛▌/GAPDH.tif]

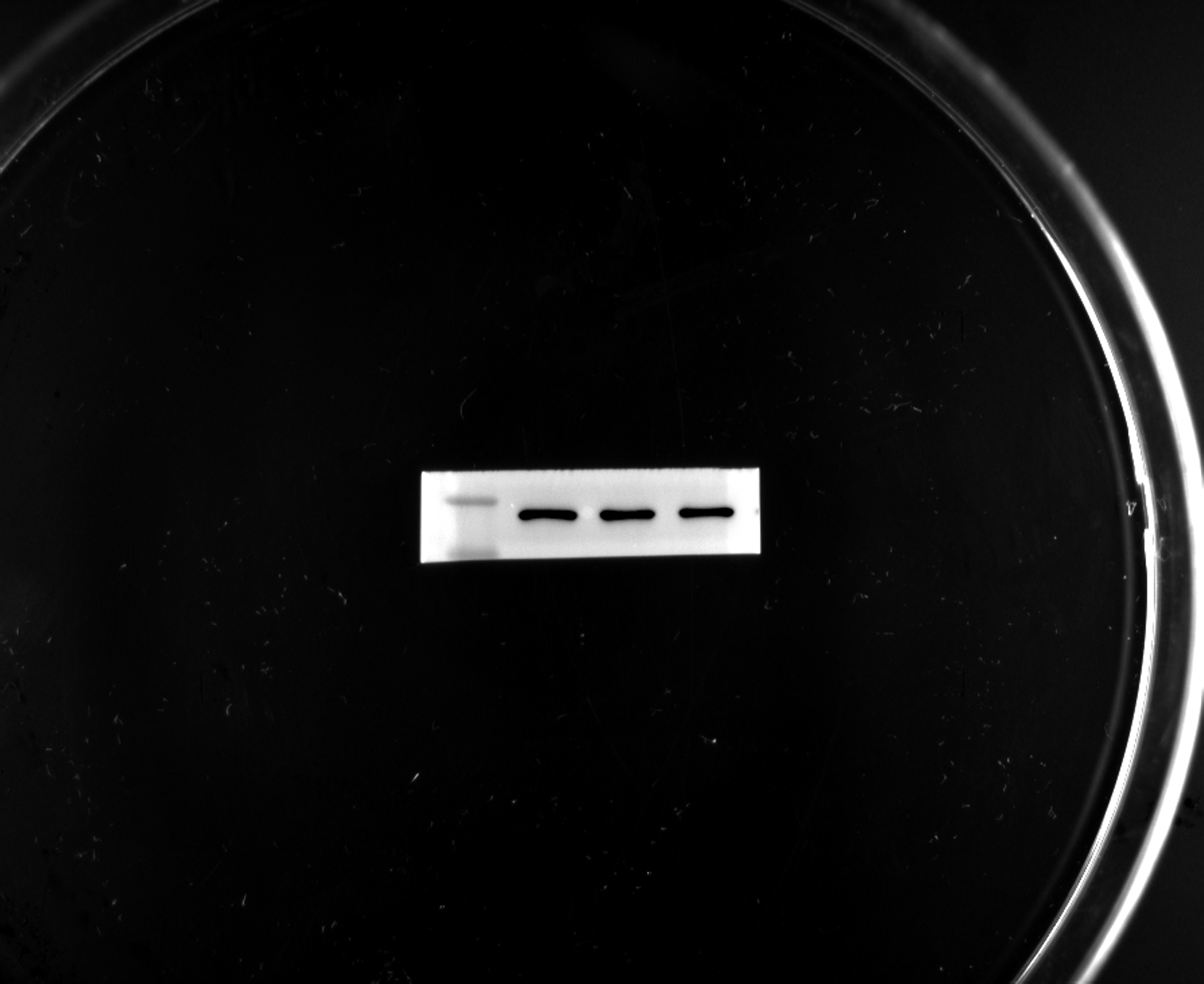

Supplement: Supplementary file 1 [file DataSheet1.zip › ╩2╛▌/GAPDH3.Tif]

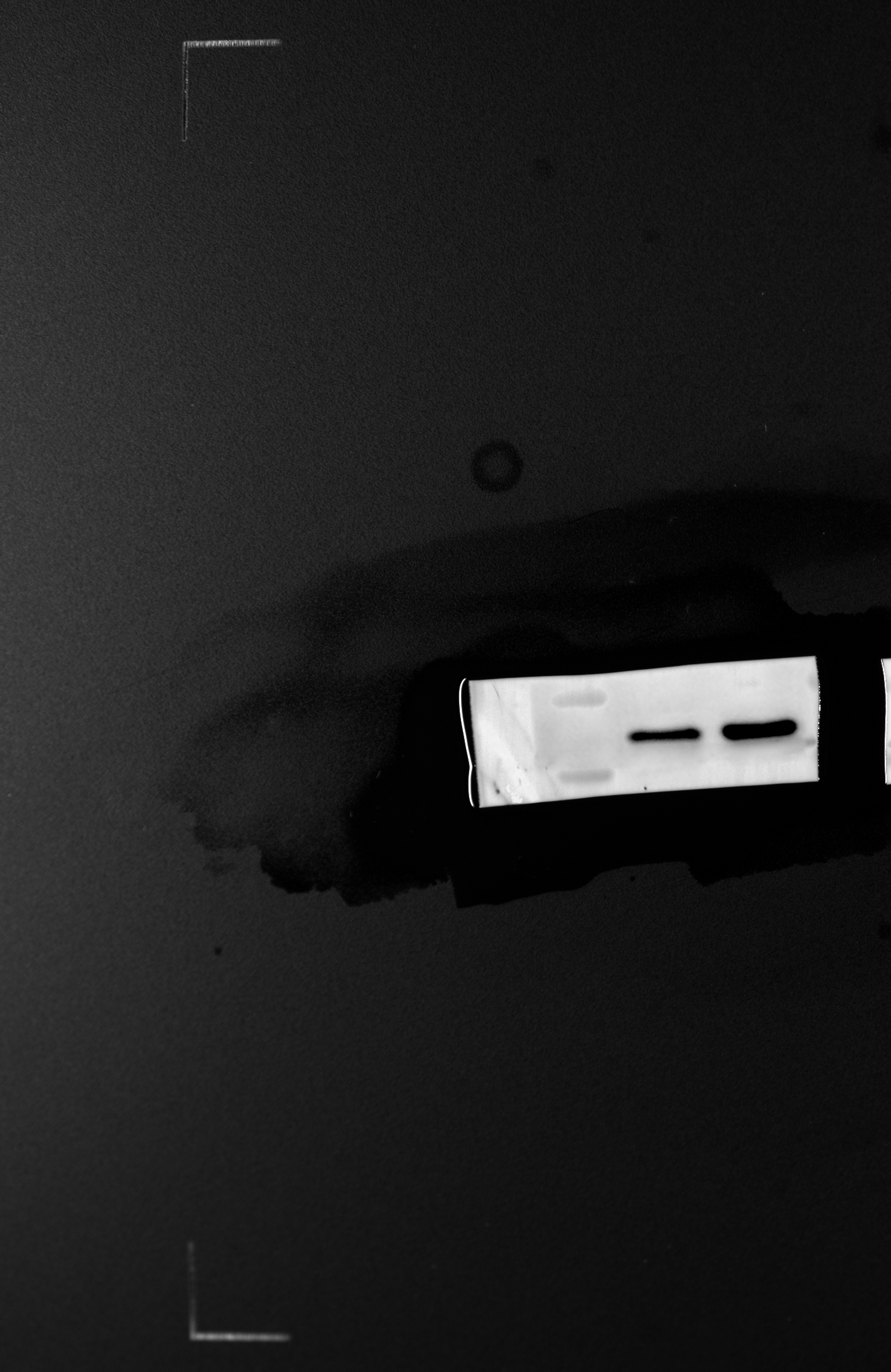

Supplement: Supplementary file 1 [file DataSheet1.zip › ╩2╛▌/PKNOX1.tif]

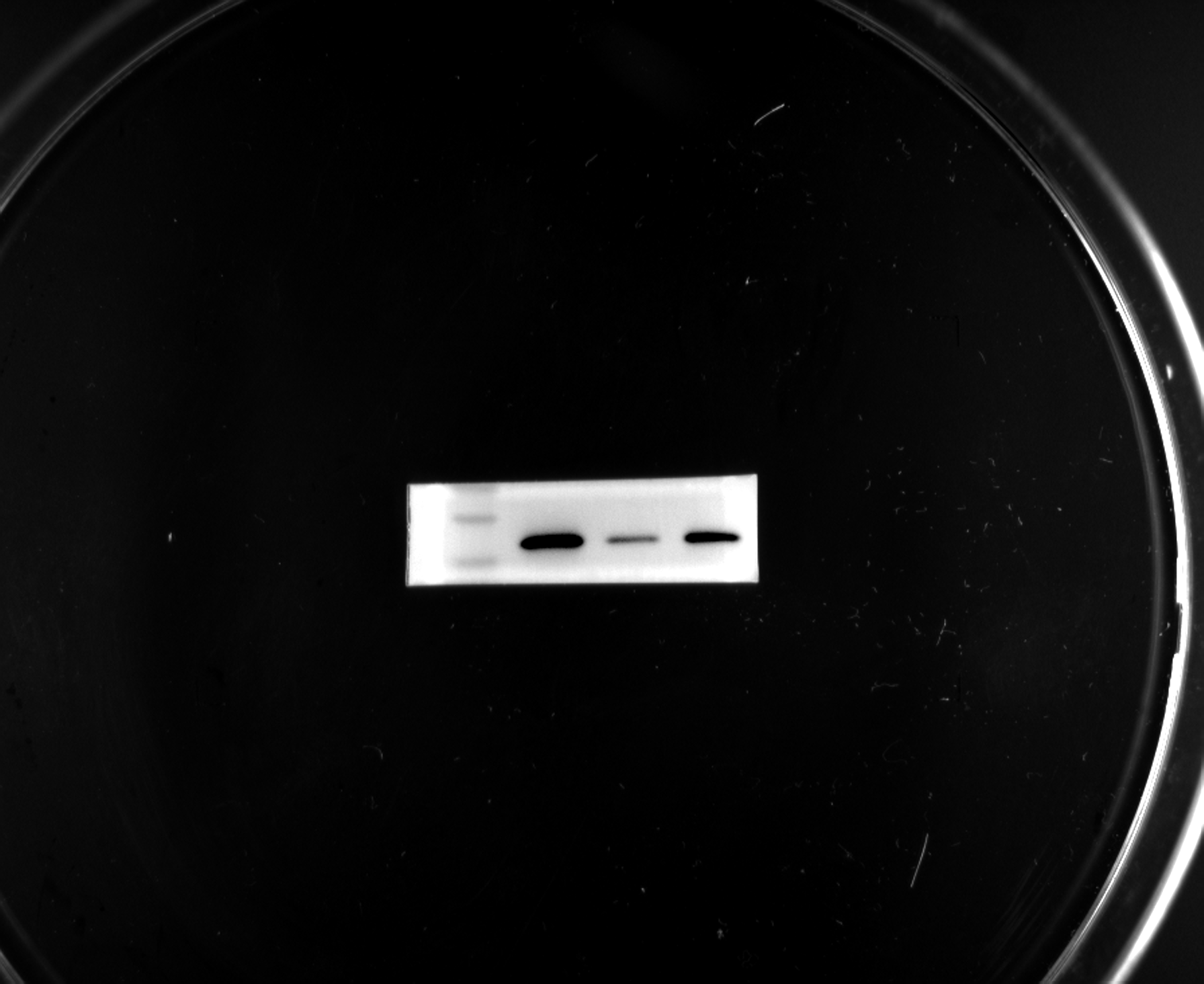

Supplement: Supplementary file 1 [file DataSheet1.zip › ╩2╛▌/PKNOX13.Tif]
